# A Bayesian Genomic Regression Model with Skew Normal Random Errors

**DOI:** 10.1534/g3.117.300406

**Published:** 2018-03-27

**Authors:** Paulino Pérez-Rodríguez, Rocío Acosta-Pech, Sergio Pérez-Elizalde, Ciro Velasco Cruz, Javier Suárez Espinosa, José Crossa

**Affiliations:** * Colegio de Postgraduados, CP 56230, Montecillos, Edo. de México; † Biometrics and Statistics Unit, International Maize and Wheat Improvement Center (CIMMYT), Apdo. Postal 6-641, 06600, Cd. de México

**Keywords:** Genomic Selection, data augmentation, asymmetric distributions, GBLUP, Ridge regression, GenPred, Shared Data Resources

## Abstract

Genomic selection (GS) has become a tool for selecting candidates in plant and animal breeding programs. In the case of quantitative traits, it is common to assume that the distribution of the response variable can be approximated by a normal distribution. However, it is known that the selection process leads to skewed distributions. There is vast statistical literature on skewed distributions, but the skew normal distribution is of particular interest in this research. This distribution includes a third parameter that drives the skewness, so that it generalizes the normal distribution. We propose an extension of the Bayesian whole-genome regression to skew normal distribution data in the context of GS applications, where usually the number of predictors vastly exceeds the sample size. However, it can also be applied when the number of predictors is smaller than the sample size. We used a stochastic representation of a skew normal random variable, which allows the implementation of standard Markov Chain Monte Carlo (MCMC) techniques to efficiently fit the proposed model. The predictive ability and goodness of fit of the proposed model were evaluated using simulated and real data, and the results were compared to those obtained by the Bayesian Ridge Regression model. Results indicate that the proposed model has a better fit and is as good as the conventional Bayesian Ridge Regression model for prediction, based on the DIC criterion and cross-validation, respectively. A computing program coded in the R statistical package and C programming language to fit the proposed model is available as supplementary material.

In genetic studies of plants or animals, it is common to find quantitative traits whose distribution is not normal; this is because the data are obtained from multiple sources or contain isolated observations (Li *et al.* 2015). Landfors *et al.* (2011) noted that it is often necessary to normalize the data to remove variation introduced during the experiment’s development. However, such standard normalization techniques are not always able to remove bias because a large number of observations are positively or negatively affected by some treatment. In addition, suitable transformation for the data may be difficult to find, and may bring further complications when estimating and interpreting the results obtained (Fernandes *et al.* 2007). To avoid transformations, different methods have been developed that are flexible enough to represent the data and reduce unrealistic assumptions (Arellano-Valle *et al.* 2005). In the genomic selection framework (GS; Meuwissen *et al.* 2001), dense molecular marker genotypes and phenotypes are used to predict the genetic values of candidates for selection. The availability of high density molecular markers of many agricultural species, together with promising results from simulations (*e.g.*, Meuwissen *et al.* 2001) and empirical studies in plants (de los Campos *et al.* 2009; de los Campos *et al.* 2010; Crossa *et al.* 2010, 2011) and animals (*e.g.*, VanRaden 2008; Weigel *et al.* 2009), are prompting the adoption of GS in several breeding programs. The parametric model for GS explains phenotypes ($y_{i}$; *i = 1,…,n*) as functions of marker genotypes ($x_{ij}$) using a linear model of the form: $y_{i}=\beta_{0}+\overset{p}{\underset{j=1}{\sum }}x_{ij}\beta_{j}+e_{i}$, where n is the number of phenotypic records, p is the number of markers, $x_{ij}\in \{0,1,2\}$ represents the number of copies of a bi-allelic marker (*e.g.*, an SNP), and $\beta_{j}$ is the additive effect of the reference allele at the *j^th^* marker, j=1,…,p. In matrix notation, the model is expressed as $y=\beta_{0}1+\mathrm{X\beta}+e$, where $y=\{y_{i}\}$, $\beta =\{\beta_{j}\}$ and $e=\{e_{i}\}$ are vectors of phenotypes, marker effects and Gaussian zero mean errors, respectively, and $X=\{x_{ij}\}$ is a matrix of marker genotypes of dimensions *n*×*p*. However, when the data are not normal, the normal regression methods generate inconsistent estimates with the natural distribution of the data and, therefore, the estimation of the conditional mean given the covariates is also inconsistent (Bianco *et al.* 2005).

With dense molecular markers, the number of markers exceeds the number of records in the reference population (**p* > >n*) and, therefore, penalized regression estimation methods and their Bayesian counterparts are commonly used. Penalized estimation methods produce regression parameter estimates that are often equivalent to posterior modes. The literature on GS offers a long list of Bayesian models whose main difference is the prior distributions assigned to marker effects, which leads to what is known as the Bayesian Alphabet (Gianola 2013; de los Campos *et al.* 2013). The above-mentioned models assume that the response follows a normal distribution. Several phenotypic traits have distributions that are skewed, for example, female flowering, male flowering, the interval between male and female flowering, categorical measurements of diseases such as ordinal scale, counting data, etc. In these cases, either a normalizing transformation for the response variable (*e.g.*, using Box-Cox transformation) or a model that deals with skew responses may be used. Varona *et al.* (2008) proposed using linear mixed models with asymmetric distributions in the residuals to tackle the problem in the context of animal breeding when pedigree information is available. Nascimento *et al.* (2017) proposed the Regularized Quantile Regression as a way to overcome the issue of non-symmetric distributions when marker information is available.

If a population is selected based on one trait (Y) and another trait of interest (O) results that exceeds (does not exceed) some threshold, then the conditional distribution of Y | O > o, for a fixed o, leads to a distribution that is skewed (Arnold and Beaver 2000), such as the skew-normal (SN) distribution, which is of particular interest in this research. This distribution is a generalization of the normal distribution (Azzalini 1985) with a shape parameter added to adopt skewed forms. It has the advantage of being mathematically tractable and shares properties with the normal distribution; for example, the density of the SN is unimodal (Genton 2004). Varona *et al.* (2008) argues that the asymmetric distributions observed for the phenotypes are the result of environmental factors and that data can be modeled using non-symmetric residual distributions.

Based on the previous considerations and motivated by the fact that a great deal of traits in plant breeding have skew normal distributed, such as flowering time in most crop species, as well categorical traits such as diseases (ordinal, binomial, or counting data), in this study we propose a general Bayesian genomic regression model for skew-normal phenotypic traits with skew-normal random errors. The model uses a stochastic representation of the response variable (Arnold and Beaver 2000) in order to ease computations and it also works in the case that when *n*>*p*. It should point out, however, that the aim of the paper is not to describe and study the causes of the skew distribution but rather we assume that the skew data are given and thus the objective is to propose a robust statistical model that deals with the skew-normal distribution of the phenotypic and residuals.

The structure of this paper is as follows. In section 2, we present the statistical models and describe the latent variable model used in the regression. In section 3, we describe a simulation experiment that is performed to evaluate the predictive power of the proposed model. In section 4, we present an application with real data; section 5 includes the discussion and concluding remarks.

## MATERIALS AND METHODS

### Statistical models

In this section we introduce the statistical models to be used in the manuscript. We begin by giving a brief review of the skew normal model. Then we introduce the concept of data augmentation and we use this concept in order to generate a skew normal random variable. After that we introduce the “centered parameterization” in the skew normal model, regression with skew random errors. Finally, we present the pior, posterior and full conditional distributions for the regression model with skew normal residuals.

#### Skew-normal model:

A continuous random variable U is said to follow the skew-normal law with shape parameter λ∈ℝ, denoted by $SN_{D}(\lambda )$ if its density function is:

$$f_{U}\left(u|\lambda \right)=2\varphi \left(u\right)\text{Φ}\left(\lambda u\right),\;u\in \mathbb{R},$$ (1)

where φ(⋅) and Φ(⋅) denote the density and cumulative distribution functions of a standard normal random variable, respectively. The subscript D indicates the use of “direct parametrization” (Azzalini 1985). Note that the skew normal distribution reduces to the normal case when λ=0.

The mean and variance of U are given by:

$$E\left(U\right)=\sqrt{\frac{2}{\pi }}\frac{\lambda }{\sqrt{1+\lambda^{2}}}$$.

$$Var\left(U\right)=1-\frac{2}{\pi }\frac{\lambda^{2}}{1+\lambda^{2}}$$.

The coefficient of skewness of U is:

$$\gamma_{1}=\frac{{\left(E\left(U\right)\right)}^{3}}{{\left\{1-{\left(E\left(U\right)\right)}^{2}\right\}}^{3/2}}$$.

If Y is a random variable defined by Y=ξ+ωU, then Y is said to have a skew-normal distribution with location (ξ), scale (ω), and shape (λ) parameters, and is denoted as $SN_{D}(\text{ξ},\text{ ω},\text{ λ})$. The density function of Y is given by:

$$f_{Y}\left(y|\xi ,\omega ,\lambda \right)=2\frac{1}{\omega }\varphi \left(\frac{y-\xi }{\omega }\right)\text{Φ}\left[\lambda \left(\frac{y-\xi }{\omega }\right)\right],\;y,\;\;\xi \in \mathbb{R},\;\omega \in \;{\mathbb{R}}^{+}$$.

It can be shown that the coefficient of skewness of Y corresponds to the skewness coefficient of U.

#### Hidden truncation:

Let *V* and *W* be two random variables whose joint distribution is given as follows:

$$\left(\begin{array}{c} V\\ W \end{array}\right)∼MN_{2}\left(\left(\begin{array}{c} 0\\ 0 \end{array}\right),\left(\begin{array}{cc} 1 & \rho \\ \rho & 1 \end{array}\right)\right)$$,

where $MN_{2}(\mu ,\Sigma )$ denotes a bivariate random variable with mean μ and variance-covariance matrix Σ and ρ∈(−1,1); the random variable *U* is defined as follows:

$$U=\{\begin{array}{l} W\;if\;V>0\\ 0\;\text{Otherwise} \end{array}$$

then U∼SN(λ), with $\lambda =\rho /\sqrt{1-\rho^{2}}$ (Arnold and Beaver 2000; Hea-Jung 2005; Liseo and Parisi 2013). The above representation allows writing an augmented likelihood function (Hea-Jung 2005; Liseo and Parisi 2013), “as if” we had observed the latent variable Z=V>0. The conditional distribution of U|Z=z is $N(\rho z,1-\rho^{2})$ and Z∼TN(0,1,0,∞), which is a truncated normal random variable with location parameter 0, scale parameter 1, lower truncation bound 0 and upper truncation bound ∞. Therefore, the joint distribution of U and Z is $f_{U|Z}\left(u|z,\rho \right)f_{Z}\left(z\right)=f_{U,Z}\;(u,z|\rho )$, that is:

$$f_{U,Z}\left(u,z|\rho \right)=\frac{1}{\sqrt{2\pi (1-\rho^{2})}}\exp\left\{-\frac{1}{2\left(1-\rho^{2}\right)}{\left(u-\rho z\right)}^{2}\right\}\times \frac{1}{\sqrt{2\pi }}\exp\left\{-\frac{1}{2}z^{2}\right\}I_{\left(0,\infty \right)}\left(z\right),\;u\in \mathbb{R}$$. (2)

Note that the density function of U can be obtained by integrating $f_{U,Z}(u,z|\rho )$ with respect to *z*; that is, $f_{U}\left(u|\rho \right)=\overset{\infty }{\underset{0}{\int }}f(u,z|\rho )dz$.

**Figure 1:**
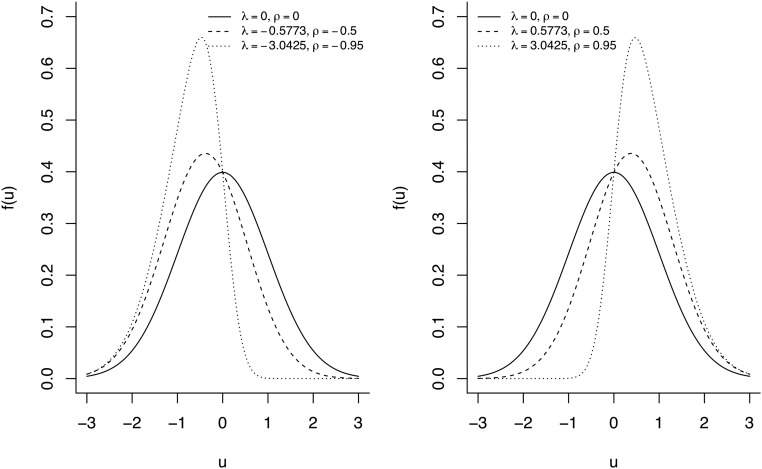
Densities of the standard skew normal distribution for different values of λ and the corresponding values for ρ, $\lambda =\frac{\;\rho }{\sqrt{1-\rho^{2}}}$.

Estimating the parameters in the direct parametrization is troublesome, so “centered parametrization” is more appropriate for parameter estimation and interpretation (Azzalini 1985; Pewsey 2000; Azevedo *et al.* 2011, among others). If $U∼SN_{D}(\lambda )$ and $\lambda =\frac{\;\rho }{\sqrt{1-\rho^{2}}}$, then $E\left(U\right)=E_{U}=\sqrt{\frac{2}{\pi }}\rho$ and $Var\left(U\right)=1-\frac{2}{\pi }\rho^{2}$ so that $S_{U}=\sqrt{Var(U)}=\sqrt{1-\frac{2}{\pi }\rho^{2}}$. Figure 1 shows the density function of *U* for several values of the shape parameter λ and the corresponding values of ρ. The random variable:

$$Y=\mu +\sigma_{e}\left(\frac{U-E_{U}}{S_{U}}\right)$$, (3)

is said to be a skew normal random variable with parameters μ∈ℝ, $\sigma_{e}>0$ and $\gamma_{1}$, where $\gamma_{1}$ is Pearson’s skewness coefficient given by γ1=2πρ3(4π−1)(1−2ρ2π)−32, and the range of $\gamma_{1}$ is (-0.99527, 0.99527). In this case, E(Y)=μ, $Var\left(Y\right)=\sigma_{e}^{2}$. The usual notation is $Y∼SN_{C}(\mu ,\sigma_{e}^{2},\gamma_{1})$.

If we consider the following transformations:

$$\begin{array}{ccc} Y & = & \mu +\sigma_{e}\left(\frac{U-E_{U}}{S_{U}}\right)\\ T & = & Z \end{array}$$, (4)

it can be shown, using Jacobians (Casella and Berger, 2002, Chapter 2), that the joint density of Y and Z is given by:

$$f_{Y,Z}\left(y,z|\mu ,\sigma_{e}^{2},\rho \right)=\frac{\zeta }{\sqrt{2\pi }}\exp\left\{-{\frac{\zeta }{2}}^{2}{\left(y-\mu -\frac{\sigma_{e}}{S_{U}}\rho z+\frac{\sigma_{e}}{S_{U}}E_{u}\right)}^{2}\right\}\times \frac{2}{\sqrt{2\pi }}\exp\left\{-\frac{1}{2}z^{2}\right\}I_{\left(0,\infty \right)}(z),$$ (5)

where $\zeta =\frac{S_{U}}{\sigma_{e}\sqrt{1-\rho^{2}}}$. Note that the density function of $Y∼SN_{C}(\mu ,\sigma_{e}^{2},\gamma_{1})$ can be obtained by integrating $f_{Y,Z}\left(y,z|\mu ,\sigma_{e},\rho \right)$ with respect to z; that is, $f_{Y}\left(y|\mu ,\sigma_{e}^{2},\gamma_{1}\right)=\overset{\infty }{\underset{0}{\int }}f_{Y,Z}\left(y,z|\mu ,\sigma_{e}^{2},\rho \right)dz$, with $\gamma_{1}=\sqrt{\frac{2}{\pi }}\rho^{3}\left(\frac{4}{\pi }-1\right){\left(1-\frac{2\rho^{2}}{\pi }\right)}^{-3/2}$. This representation is very convenient, because it allows us to write an augmented likelihood function (Hea-Jung 2005; Liseo and Parisi 2013), “as if” we had observed the latent value z. The density function of the skew normal random variable under “centered parametrization” is a complicated function that was given by Azevedo *et al.* (2011):

$$f_{Y}\left(y|\mu ,\sigma_{e}^{2},\gamma_{1}\right)=\frac{1}{\sqrt{\sigma_{e}^{2*}}}\;\varphi \left(\frac{y-\mu^{*}}{\sqrt{\sigma_{e}^{2*}}}\right)\text{Φ}\left[\lambda^{*}\left(\frac{y-\mu^{*}}{\sqrt{\sigma_{e}^{2*}}}\right)\right]$$,

where $\mu^{*}=\mu -s\gamma_{1}^{\frac{1}{3}}$, $\sigma_{e}^{2*}=\sigma_{e}^{2}\times \left(1+s^{2}\gamma_{1}^{2/3}\right)$, $\lambda^{*}=\frac{s\gamma_{1}^{1/3}}{\sqrt{r^{2}+s^{2}\gamma_{1}^{2/3}(r^{2}-1)}}$ with $r=\sqrt{2/\pi }$, $s={\left(\frac{2}{4-\pi }\right)}^{1/3}$.

#### Regression with skew normal random errors:

Azzalini and Capitanio (1999) and Rusell and González (2002) proposed a simple linear regression model where the error terms are independent and identically distributed as $SN_{D}(0,\text{ ω},\text{ λ})$. The proposed model is:

$$y_{i}=\beta_{0}+\beta_{1}x_{i}+e_{i}$$;

from the properties of the skew normal distribution, it follows that $y_{i}∼SN_{D}(\beta_{0}+\beta_{1}x_{i},\omega ,\lambda )$. The model can be easily extended to include more covariates; that is:

$$y_{i}=\beta_{0}+\overset{p}{\underset{j=1}{\sum }}x_{ij}\beta_{j}+e_{i}=\beta_{0}+x_{i}^{t}\beta +e_{i}$$.

Azzalini and Capitanio (1999) and Rusell and González (2002) used the maximum likelihood method to estimate the parameters in the model. These ideas can be extended to the case of errors that are independent and identically distributed as $SN_{C}(0,\;\sigma_{e}^{2},{\text{ γ}}_{1})$.

#### Bayesian regression with skew normal random errors (BSN):

Let $y_{i}∼SN_{c}\left(\beta_{0}+x_{i}^{t}\beta ,\sigma_{e}^{2},\gamma_{1}\right),\;i=1,\ldots ,n.$ Then, the likelihood function is given by:

$$p\left(y|\beta_{0},\beta ,\sigma_{e}^{2},\;\gamma_{1}\right)=\overset{n}{\underset{i=1}{\prod }}SN_{C}(y_{i}|{\text{β}}_{0}+x_{i}^{t}\beta ,\sigma_{e}^{2},\gamma_{1})$$.

Let $\theta ={\left(\beta_{0},\beta^{t},\sigma_{e}^{2},\gamma_{1}\right)}^{t}$ and p(θ|Ω) the prior distribution of θ and Ω a set of hyper-parameters that index the prior distributions. Then, by Bayes’ theorem, the joint posterior distribution of p(θ|y) is as follows:

$$\begin{array}{c} p\left(\theta |y\right)\propto p\left(y|\beta_{0},\beta ,\sigma_{e}^{2},\;\gamma_{1}\right)p(\theta |\Omega )\\ =\overset{n}{\underset{i=1}{\prod }}SN_{C}(y_{i}|{\text{β}}_{0}+x_{i}^{t}\beta ,\sigma_{e}^{2},\gamma_{1})\;p(\theta |\Omega ). \end{array}$$

Neither the joint posterior distribution nor the full conditional distributions of the parameters of interest have a closed form; therefore, the implementation of this model within the Bayesian framework is computing intensive. We propose using hidden truncation together with two standard MCMC techniques in Bayesian analysis: (i) Gibbs Sampling (Geman and Geman 1984) and (ii) Random Walk Metropolis Sampling to alleviate some of the computing burden.

#### Prior, posterior and full conditional distributions:

Consider the joint distribution of Y and Z given in (5). In the regression context, we set $\mu_{i}=\beta_{0}+x_{i}^{t}\beta$; then the augmented likelihood function is:

$$\begin{array}{c} p\left(y,z|\beta_{0},\beta ,\sigma_{e}^{2},\rho \right)=\overset{n}{\underset{i=1}{\prod }}f_{Y_{i},Z_{i}}\left(y_{i},z_{i}|\mu_{i},\sigma_{e}^{2},\rho \right)\\ \propto \overset{n}{\underset{i=1}{\prod }}\zeta \text{exp}\left\{-\frac{1}{2}\zeta^{2}{\left(y_{i}-\mu_{i}-\frac{\sigma_{e}}{S_{U}}\rho z_{i}+\frac{\sigma_{e}}{S_{U}}E_{u}\right)}^{2}-\frac{1}{2}z_{i}^{2}\right\}I_{\left(0,\infty \right)}\left(z_{i}\right). \end{array}$$ (6)

In order to fully specify the Bayesian model, prior distributions for the unknown parameters must be defined. Let $\beta |\sigma_{\beta }^{2}∼MN_{p}(0,\sigma_{\beta }^{2}I)$; for ρ, based on the following transformation ρ=1−2B, where $B∼Beta\left(a_{0},b_{0}\right),$ the prior for ρ is denoted as $p(\rho |a_{0},b_{0})$, and depending on the hyper-parameters $a_{0}$ and $b_{0}$, it can lead to a rich variety of shapes, just as in the case of the Beta distribution. For the intercept, the prior distribution is $\beta_{0}|\sigma_{\beta_{0}}^{2}∼N(0,\sigma_{\beta_{0}}^{2})$; for the scale parameter, a scaled inverted chi-squared prior distribution was used, that is, $\sigma_{e}^{2}|df_{e},S_{e}∼\chi^{-2}\left(df_{e},S_{e}\right),$ and finally $\sigma_{\beta }^{2}|df_{\beta },S_{\beta }∼\chi^{-2}(df_{\beta },S_{\beta })$ (see Sorensen and Gianola, 2002, p. 85, for details about the parametrization used in this paper). The joint prior distribution is

$$p\left(\beta_{0},\beta ,\sigma_{e}^{2},\sigma_{\beta }^{2},\rho |\Omega \right)=p(\beta_{0}|\sigma_{\beta_{0}}^{2})p(\beta |\sigma_{\beta }^{2})p(\sigma_{\beta }^{2}|df_{\beta },S_{\beta })p(\sigma_{e}^{2}|df_{e},S_{e})p(\rho |a_{0},b_{0})$$. (7)

By combining equations 6 and 7 throught the Bayes’ theorem, the posterior distribution of $p\left(\beta_{0},\beta ,\sigma_{e}^{2},\sigma_{\beta }^{2},\rho |data\right)$ is given by:

$$\begin{array}{c} p\left(\beta_{0},\beta ,\sigma_{e}^{2},\sigma_{\beta }^{2},\rho |data\right)\propto \left\{\overset{n}{\underset{i=1}{\prod }}\zeta \exp\left\{-\frac{1}{2}\zeta^{2}{\left(y_{i}-\mu_{i}-\frac{\sigma_{e}}{S_{U}}\rho z_{i}+\frac{\sigma_{e}}{S_{U}}E_{u}\right)}^{2}-\frac{1}{2}z_{i}^{2}\right\}I_{\left(0,\infty \right)}(z_{i})\right\}\\ \times N\left(\beta_{0}|0,\sigma_{\beta_{0}}^{2}\right)MN_{p}\left(\beta |0,\sigma_{\beta }^{2}I\right)\chi^{-2}\left(\sigma_{e}^{2}|S_{e},df_{e}\right)\;\chi^{-2}\left(\sigma_{\beta }^{2}|S_{\beta },df_{\beta }\right)\\ \times {\left(\frac{1-\rho }{2}\right)}^{a_{0}-1}{\left(1-\frac{1-\rho }{2}\right)}^{b_{0}-1}I_{\left(-1,1\right)}\left(\rho \right). \end{array}$$ (8)

The full conditional distributions of the parameters are given in Appendix A, which are the inputs for the Gibbs and the Random Walk Metropolis Samplers. In Appendix B, some pragmatical rules to elicitate values for the hyper-parameters $\sigma_{\beta_{0}}^{2},\;S_{e},df_{e}$, $S_{\beta },df_{\beta },\;a_{0}$ and $b_{0}$, are given. The rules for setting $S_{e},df_{e}$, $S_{\beta },df_{\beta }$ are based on those given by de los Campos *et al.* (2013) and Pérez and de los Campos (2014). In this paper, we set the hyper-parameters as follows: $\sigma_{\beta_{0}}^{2}=1\times 10^{6}$, $df_{e}=df_{\beta }=5$,$\;S_{e}=0.5\times V_{y}\times \left(df_{e}+2\right)$, $S_{\beta }=0.5\times V_{y}\times \frac{\left(df_{\beta }+2\right)}{MSx}$, where $V_{y}$ is the sample phenotypic variance and $MSx=\frac{1}{n}\overset{n}{\underset{i=1}{\sum }}\overset{p}{\underset{j=1}{\sum }}x_{ij}^{2}$. We set $\sigma_{\beta_{0}}^{2}=1\times 10^{6}$ in order to reduceshrinkage and because in practice it mimics a non informative but proper distribution. To sample from the full conditionals of ρ and $\sigma_{e}^{2}$, we implemented a Random Walk Metropolis Sampler whose parameters are tuned so that the acceptation rate is about 0.23 (see Appendix A for details).

The BSN can be re-parametrized by replacing $x_{i}^{t}\beta$ with $t_{i}=x_{i}^{t}\beta$; if the prior distribution of marker effects is normal with mean 0 and variance $\sigma_{\beta }^{2}$, then the prior of t is $t∼{MN}_{n}(0,\sigma_{\beta }^{2}\mathrm{XX}’)$, which leads to a G-BLUP model (see de los Campos *et al.* 2013, for details about G-BLUP) but with skew normal residuals, that is, $y_{i}=\beta_{0}+t_{i}+e_{i}$ or, in matrix notation, $y=\beta_{0}1+t+e$, which is a skew linear mixed model, a particular case of the model proposed by Arellano-Valle *et al.* (2005, 2007), who relaxed all normality assumptions in a standard mixed model.

#### Bayesian ridge regression With random normal errors (BRR):

Regression with random normal errors is a special case of the proposed model when ρ=0. The model is widely used in the GS selection literature (*e.g.*, de los Campos *et al.* 2013). In the GS context, the model is given by:

$$y_{i}=\beta_{0}+\overset{p}{\underset{j=1}{\sum }}\beta_{j}x_{ij}+e_{i}$$,

where $e_{i}’s$ are independent and identically distributed as $N(0,\sigma_{e}^{2})$.

The prior distributions for the unknown are: $\beta |\sigma_{\beta }^{2}∼MN_{p}(0,\sigma_{\beta }^{2}I)$, $\beta_{0}|\sigma_{\beta_{0}}^{2}∼N(0,\sigma_{\beta_{0}}^{2})$ for the scale parameter, a scaled inverted chi-squared distribution, that is, $\sigma_{e}^{2}|df_{e},S_{e}∼\chi^{-2}\left(df_{e},S_{e}\right)$ and finally $\sigma_{\beta }^{2}|df_{\beta },S_{\beta }∼\chi^{-2}(df_{\beta },S_{\beta })$.

The joint prior distribution is

$$p\left(\beta_{0},\beta ,\sigma_{e}^{2},\sigma_{\beta }^{2}\right)=p(\beta_{0}|\sigma_{\beta_{0}}^{2})p(\beta |\sigma_{\beta }^{2})p(\sigma_{\beta }^{2}|df_{\beta },S_{\beta })p(\sigma_{e}^{2}|df_{e},S_{e})$$. (9)

Thus, the posterior distribution of $p\left(\beta_{0},\beta ,\sigma_{e}^{2},\sigma_{\beta }^{2}|data\right)$ is

$$\begin{array}{c} p\left(\beta_{0},\beta ,\sigma_{e}^{2},\sigma_{\beta }^{2}|data\right)\propto \left\{\overset{n}{\underset{i=1}{\prod }}N(y_{i}|\mu_{i},\sigma_{e}^{2})\right\}\\ \times N\left(\beta_{0}|0,\sigma_{\beta_{0}}^{2}\right)MN_{p}\left(\beta |0,\sigma_{\beta }^{2}I\right)\chi^{-2}\left(\sigma_{e}^{2}|S_{e},df_{e}\right)\;\chi^{-2}\left(\sigma_{\beta }^{2}|S_{\beta },df_{\beta }\right). \end{array}$$

The required full conditional distributions of the parameters for implementing a Gibbs sampler can be found elsewhere (*e.g.*, de los Campos *et al.* 2013). We set the hyper-parameters using the same rules as in the BSN model. The BRR model can be fitted easily using the BGLR statistical package (Pérez and de los Campos 2014).

### Monte Carlo Simulation

In this section, we use simulated data using marker genotypes from a wheat dataset made publicly available by Crossa *et al.* (2010). The dataset includes genotypic information for 599 wheat lines which were genotyped for 1279 DArT markers coded as 0 and 1. We simulated the phenotypes using the following additive genetic model:

$$y_{i}=\beta_{0}+\overset{1279}{\underset{j=1}{\sum }}x_{ij}\beta_{j}+e_{i},\;i=1,\ldots ,n$$, (9)

where $e_{i}∼SN_{C}(0,1.5^{2},\gamma_{1})$, with $\gamma_{1}=\sqrt{\frac{2}{\pi }}\rho^{3}\left(\frac{4}{\pi }-1\right){\left(1-\frac{2\rho^{2}}{\pi }\right)}^{-3/2}$, ρ∈{0,.5,.75,.90,.95,.99}, which leads to different degrees of skewness. The intercept parameter, $\beta_{0},$ was set equal to 3; 10 marker effects were sampled from a normal distribution with mean 0 and variance 0.5/10 (Pérez and de los Campos 2014), and the rest were set equal to 0, that is:

$$\beta_{j}=\{\begin{array}{c} \begin{array}{cc} N(0,0.5/10) & \text{if}\;j\in \{63,190,\;317,444,571,698,825,952,1079,1206\},\\ 0 & \text{otherwise} \end{array} \end{array}$$

The idea here is to verify, through simulation, whether the proposed model works satisfactorily. We therefore obtained point estimates for $\beta_{0}$, β, $\sigma_{e}^{2}$ and ρ. We also fitted the Bayesian Ridge Regression model and compared the estimates of regression coefficients, predictions and estimates of genetic values of both models. Let $\hat{\beta }$ be the vector of posterior means for regression coefficients. Pearson’s correlation between the observed (y) and predicted values (${\hat{\beta }}_{0}1+X\hat{\beta }$) is a goodness-of-fit measure; Pearson’s correlation between the “true” genetic values (Xβ) and the predicted values ($X\hat{\beta }$) is a measure of how well the genetic values are estimated; finally, Pearson’s correlation between the “true” marker effects (β) and the estimated effects ($\hat{\beta }$) is a measure that indicates how good a model is at uncovering marker effects (de los Campos *et al.* 2009). We also computed the effective number of parameters (pD) and deviance information criterion (DIC) for the two fitted models (see Spiegelhalter *et al.* 2002, for more details).

The algorithm used in this simulation experiment is described briefly below.

1. Set $\beta_{0}$, β, $\sigma_{e}^{2}$ and ρ.
2. Simulate the phenotypes using equation (9).
3. Fit the regression model with skew normal random errors and obtain point estimates for $\beta_{0}$, β, $\sigma_{e}^{2}$ and ρ, that is, ${\hat{\beta }}_{0SN}$, ${\hat{\beta }}_{SN}$, ${\hat{\sigma }}_{eSN}^{2}$ and $\hat{\rho }$. The point estimates correspond to the posterior means of the posterior distribution of the parameters of interest.
4. Fit the Bayesian Ridge Regression model and obtain point estimates for $\beta_{0}$, β, $\sigma_{e}^{2}$, that is, ${\hat{\beta }}_{0N}$, ${\hat{\beta }}_{N}$, ${\hat{\sigma }}_{eN}^{2}$.
5. Compute the correlation between observed and predicted phenotypes, “true” and predicted genetic values, and “true” and estimated regression coefficients with both regression models.
6. Compute the effective number of parameters (pD) and deviance information criterion (DIC) for the two fitted models.
7. Repeat steps 1 to 5 one hundred times and obtain the averages of correlations, intercept ($\beta_{0}$), $\sigma_{e}^{2}$ and ρ.

### Application to real data

**Figure 2:**
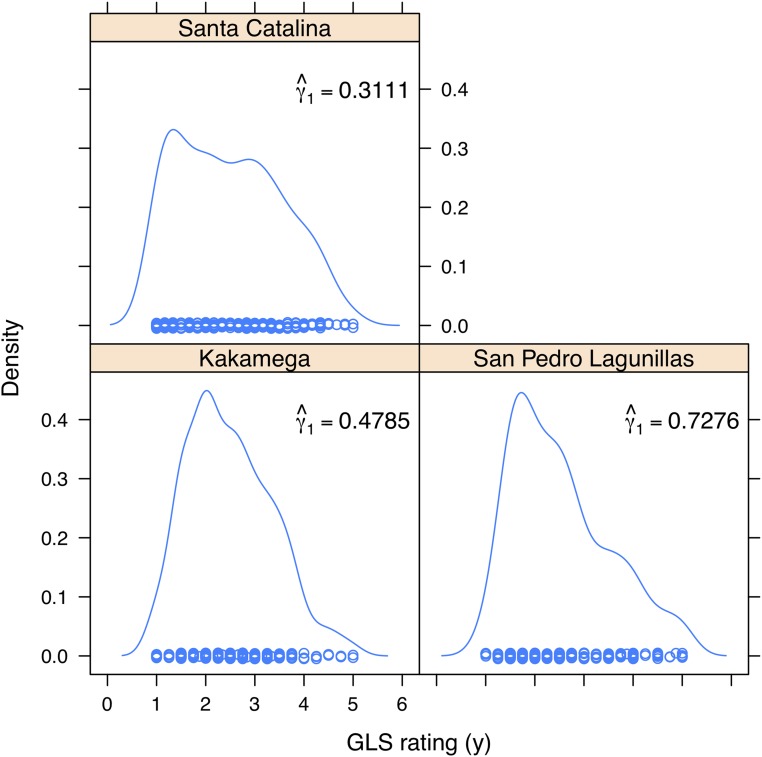
Density plot for Gray Leaf Spot (GLS) rating (disease resistance), at each site: Kakamega (Kenya), San Pedro Lagunillas (Mexico) and Santa Catalina (Colombia).

This dataset is from the Drought Tolerance Maize (DTMA) project of CIMMYT’s Global Maize Program (http://www.cimmyt.org). The dataset comes from a large study aimed at detecting chromosomal regions affecting drought tolerance. The genotypic data consist of information from 300 tropical inbred lines that were genotyped using 1,152 SNPs (Single Nucleotide Polymorphisms). The analyzed trait is Gray Leaf Spot (GLS) caused by the fungus *Cercospora zeae-maydis*, which was evaluated at three different sites, Kakamega (Kenya), San Pedro Lagunillas (Mexico) and Santa Catalina (Colombia) (see Supporting information). Crossa *et al.* (2011) analyzed a subset of these data; the response variable was transformed using Box-Cox transformation (Box and Cox 1964). Figure 2 shows density plots for GLS rating at the three sites. Kernel density was estimated using a Gaussian kernel, and the bandwidth for the kernel was estimated according to Venables and Ripley (2002). Figure 2 also shows the sample skewness index, ${\hat{\gamma }}_{1}=m_{3}/s^{3}$, where $m_{3}=n^{-1}\overset{n}{\underset{i=1}{\sum }}{\left(y_{i}-\bar{y}\right)}^{3}$, $\bar{y}$ is the sample mean and s is the sample standard deviation (see Joanes and Gill 1998); in the three cases, the distribution is skewed to the right, so most of the distribution is concentrated around small values of the response variable.

We propose using the regression model with skew normal random errors to predict disease resistance. We fitted two models: (1) the standard Bayesian Ridge Regression, where the errors $e_{i}∼NIID\left(0,\sigma_{e}^{2}\right),\;i=1,\ldots ,n$, where “NIID” stands for “normally, independent and identically distributed”; and (2) the proposed model with skew normal random errors. The Bayesian Ridge Regression was fitted using the BGLR package (Pérez and de los Campos 2014), whereas the proposed model was fitted using the algorithm described in Appendix A. The models were first fitted using the full data, and subsequently 100 random partitions with 80% of observations in the training set and 20% of observations in the testing set were generated. The two models were fitted for each of these random partitions; then the phenotypes of the individuals in the testing set were predicted and the ability of each model to make predictions was evaluated using Pearson’s correlation between observed and predicted values. Inferences for each fit were based on 100,000 samples obtained after discarding 50,000 samples that were taken as burn-in. Convergence was checked by inspecting trace plots of the parameters.

### Data and program availability

The data and programs are available as File S1 which corresponds to a compressed zip folder. The zip folder also contains a description of the data and commands to read it into the R statistical software.

## RESULTS

### Monte Carlo Simulation

Table 1 shows point estimates for $\beta_{0}$, β, $\sigma_{e}^{2}$ and ρ for the BSN and BRR models for different values of ρ. It also shows an estimate of $\theta =\sigma_{e}^{2}/\sigma_{\beta }^{2}$, a regularization parameter that is widely used in Bayesian Ridge Regression. Higher values of the parameter are associated with more shrinkage; note that the estimates of $\sigma_{e}^{2}$ are very similar in both models, so small values of $\sigma_{\beta }^{2}$ could be associated with more precise estimates of β. It is also clear from this table that the point estimates for $\beta_{0}$ and $\sigma_{e}^{2}$ are very close to the real values used in the simulation. The correlation between observed and predicted values and the mean squared error is quite similar for both models and there is no clear winner. Finally, the algorithm is not able to estimate precisely the parameter ρ for distributions that are slightly asymmetric.

**Table 1:** Point estimates, standard deviations for $\beta_{0}$, β, $\sigma_{e}^{2}$, correlations between observed and predicted values and MSE of predictions. Phenotypes were simulated under model (9) with ρ∈{0,.5,.75,.90,.95,.99} and then regression models with skew normal (BSN) and normal errors (BRR) were fitted

Model	${\hat{\beta }}_{0}$	${\hat{\sigma }}_{e}^{2}$	$\;{\hat{\sigma }}_{\beta }^{2}$	$\hat{\theta }$	$Cor(y,\hat{y})$	MSE
$\text{ρ}=0,\;\hat{\rho }=0.016\;\left(0.207\right)$						
BSN	3.075 (0.854)	2.257 (0.052)	0.003 (0.001)	833.72	0.479	2.441
BRR	3.113 (0.975)	2.207 (0.155)	0.003 (0.001)	627.33	0.531	3.036
$\rho =0.5,\hat{\rho }=0.075\;(0.270)$						
BSN	3.009 (0.771)	2.218 (0.047)	0.003 (0.001)	803.35	0.648	3.274
BRR	2.991 (0.905)	2.167 (0.133)	0.003 (0.001)	602.89	0.667	2.714
$\rho =0.75,\;\hat{\rho }=0.329\;(0.261)$						
BSN	2.972 (0.714)	2.210 (0.048)	0.003 (0.001)	816.71	0.442	2.219
BRR	2.945 (0.828)	2.168 (0.139)	0.003 (0.001)	614.19	0.506	2.154
$\;\rho =0.90,\;\hat{\rho }=0.841\;(0.115)$						
BSN	3.094 (0.821)	2.219 (0.054)	0.003 (0.001)	833.48	0.648	3.112
BRR	3.120 (0.858)	2.175 (0.155)	0.003 (0.001)	621.59	0.676	2.639
$\rho =0.95\;\;\hat{\rho }=0.943\;(0.023)$						
BSN	3.055 (0.942)	2.270 (0.061)	0.003 (0.001)	872.98	0.662	1.676
BRR	3.067 (0.900)	2.196 (0.169)	0.003 (0.001)	631.79	0.696	1.642
$\rho =0.99\;\hat{\rho }=0.987\;(0.008)$						
BSN	2.830 (1.280)	2.167 (0.056)	0.003 (0.001)	668.16	0.578	2.593
BRR	2.890 (0.878)	2.165 (0.153)	0.004 (0.001)	606.84	0.631	2.893

$\hat{\theta }={\hat{\sigma }}_{e}^{2}/{\hat{\sigma }}_{\beta }^{2}$.

Table 2 shows the effective number of parameters pD, the deviance information criterion (*DIC*), the correlation between “true” and estimated marker effects and the correlation between “true” and estimated signals. The table also shows that in general the pD and the *DIC* (small is better) favored the BSN model. The correlation between “true” and estimated marker effects is slightly better for BSN and the difference between the two models becomes clearer as ρ increases. The same pattern is observed for the correlations between true and estimated genetic signals.

**Table 2:** True and estimated posterior mean of ρ, effective number of parameters (pD), deviance information criterion (*DIC*), correlations between “true” and estimated marker effects and correlations between “true” and estimated genetic signals; standard deviations in parentheses. Phenotypes were simulated under model (9) with ρ∈{0,.5,.75,.90,.95,.99} and then regression models with skew normal (BSN) and normal errors (BRR) were fitted

Model	pD	DIC	$Cor(\beta ,\;\hat{\beta })$	$Cor(\mathrm{X\beta},\;X\hat{\beta })$
$\rho =0,\;\hat{\rho }=0.016\;(0.207)$				
BSN	40.794	2206.112	0.192 (0.046)	0.697 (0.116)
BRR	59.573	2212.001	0.193 (0.049)	0.689 (0.115)
$\rho =0.5,\;\hat{\rho }=0.075\;(0.270)$				
BSN	80.469	2279.974	0.207 (0.049)	0.718 (0.119)
BRR	91.548	2279.706	0.207 (0.050)	0.714 (0.117)
$\rho =0.75,\;\hat{\rho }=0.329\;(0.261)$				
BSN	41.996	2262.930	0.194 (0.051)	0.717 (0.104)
BRR	57.826	2267.114	0195 (0.052)	0.708 (0.104)
$\rho =0.90,\;\hat{\rho }=0.841\;(0.115)$				
BSN	76.978	2218.017	0.203 (0.049)	0.718 (0.114)
BRR	96.31	2238.787	0.198 (0.052)	0.706 (0.115)
$\rho =0.95\;\;\hat{\rho }=0.943\;(0.023)$				
BSN	93.687	2144.77	0.203 (0.046)	0.734 (0.109)
BRR	102.345	2174.32	0.191 (0.047)	0.707 (0.116)
$\rho =0.99,\;\hat{\rho }=0.987\;(0.008)$				
BSN	85.465	2151.505	0.216 (0.055)	0.747 (0.098)
BRR	83.422	2276.08	0.196 (0.052)	0.703 (0.109)

### Application to real data

#### Full data:

Table 3 shows estimates of the posterior means of parameters $\sigma_{e}^{2}$, $\sigma_{\beta }^{2}$ and ρ, as well as the effective number of parameters (pD) and the deviance information criterion (*DIC*). From Table 3 it is clear that the estimation of marker effects is more precise for the BSN model than for the BRR model; the pD and the *DIC* also favored the BSN model. The estimated ρ parameter also supports the assumption that the skew normal random error is correct, and that the point estimate is not around 0, except in the case of San Pedro Lagunillas.

**Table 3:** Estimates of posterior means of parameters $\sigma_{e}^{2}$, $\sigma_{\beta }^{2}$ and ρ from the full-data analysis of Kakamega, San Pedro Lagunillas and Santa Catalina for Gray Leaf Spot in 300 tropical inbred maize lines and 1,152 SNPs; standard deviations in parentheses

Site	Parameter						
	Model	${\hat{\sigma }}_{e}^{2}$	${\hat{\sigma }}_{\beta }^{2}$	$\hat{\theta }$	pD	DIC	$\hat{\rho }$
Kakamega	BSN	0.498 (0.0725)	0.00032 (9e-05)	1726.551 (723.794)	61.257	586.361	0.981 (0.021)
	BRR	0.425 (0.073)	0.00053 (0.00014)	901.913 (391.380)	97.367	629.15	—
San Pedro Lagunillas	BSN	0.369 (0.079)	0.00093 (0.00019)	425.973 (173.541)	126.833	602.752	0.376 (0.550)
	BRR	0.331 (0.069)	0.00104 (0.00019)	339.114 (125.014)	143.06	597.852	—
Santa Catalina	BSN	0.518 (0.092)	0.00046 (0.00015)	1331.033 (785.158)	50.072	555.512	0.9226 (0.227)
	BRR	0.404 (0.070)	0.00075 (0.00016)	574.862 (199.984)	112.447	595.027	—

$\hat{\theta }={\hat{\sigma }}_{e}^{2}/{\hat{\sigma }}_{\beta }^{2}.$

**Figure 3:**
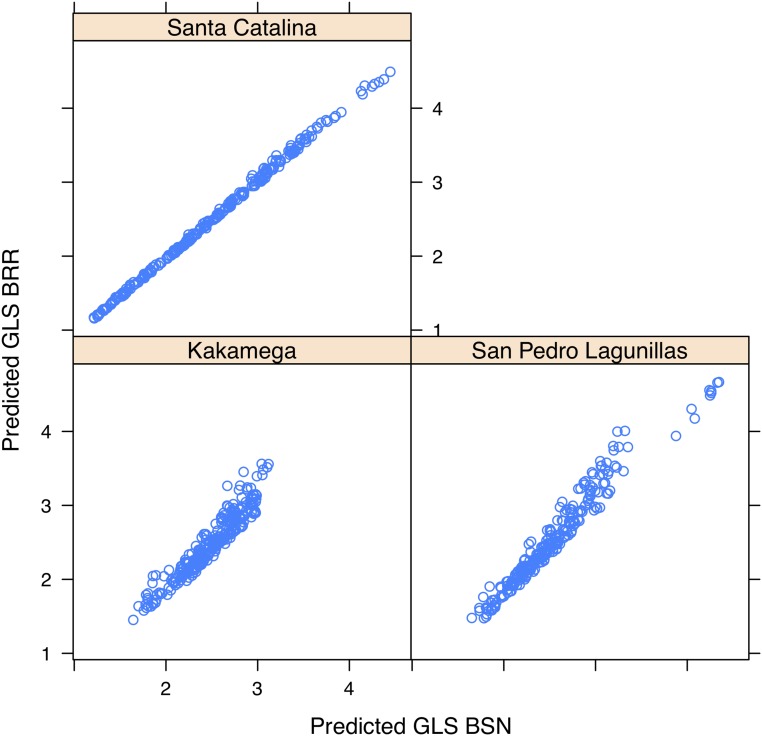
Scatterplot of predicted Gray Leaf Spot (GLS) obtained when fitting the BSN model and the BRR model. In the three cases considered, the Pearson’s correlation between predictions was higher than 0.95.

Figure 3 shows scatterplots of the predicted GLS using the BSN and BRR models. As expected, Pearson’s correlation between both predictions is very high (higher than 0.95). That implies that even when the data are skewed, if a BRR model is fitted in order to obtain candidates for selection, we can expect to obtain about the same individuals. Two models were fitted for each site by BRR and BSN.

#### Cross-validation:

**Figure 4:**
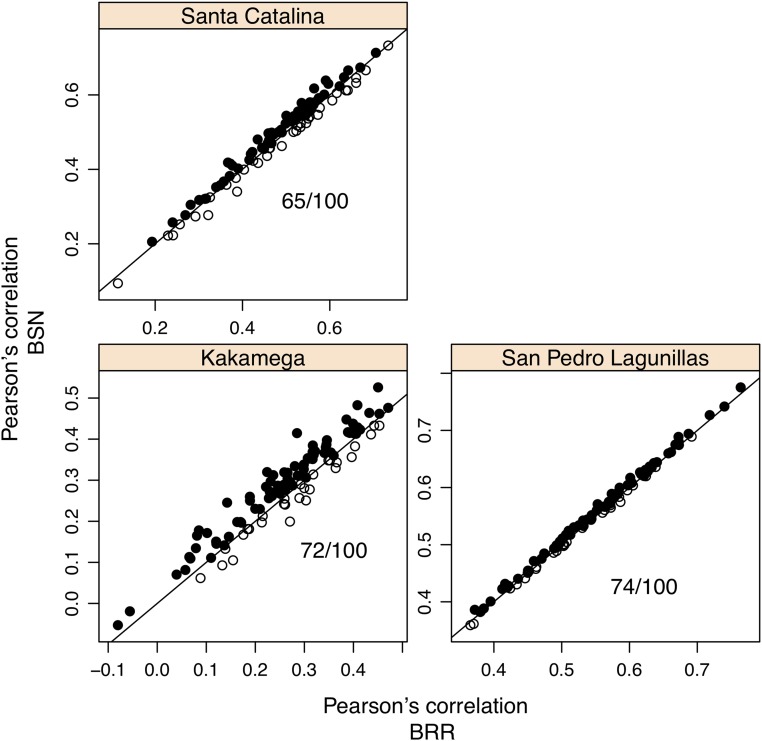
Plots of the predictive correlation for each of the 100 cross-validations and 3 locations. When the best model is BSN, this is represented by a filled circle, and when the best model is BRR, this is represented by an open circle. The number of times that Pearson’s correlation in BSN is better than Pearson’s correlation in BRR is also shown in the plots.

**Figure 5:**
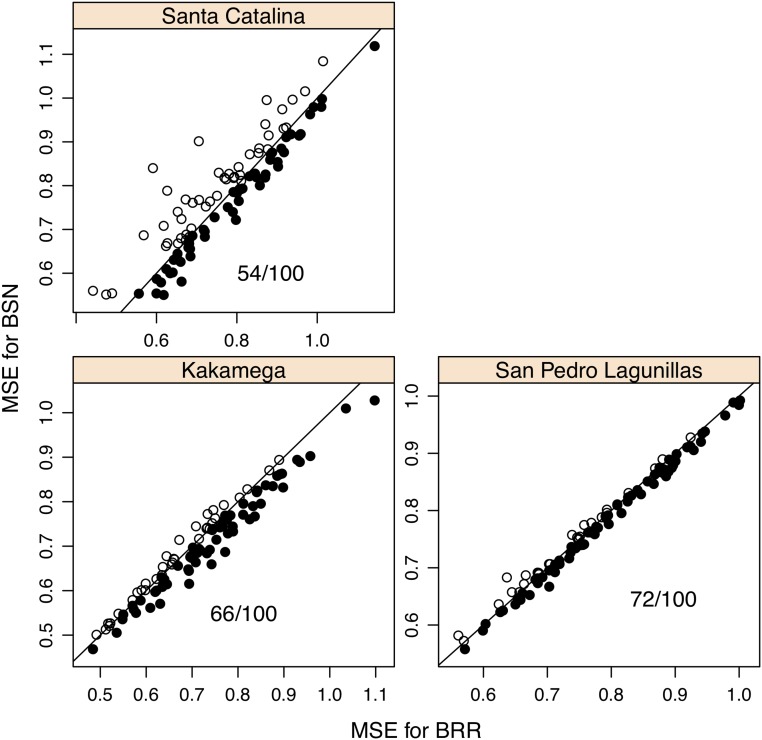
Plots of the mean squared error (MSE) in the testing set for each of the 100 cross-validations and 3 locations. When the MSE in BSN is smaller than the MSE in BRR, this is represented by an open circle and when the MSE in BRR is bigger than the MSE in BSN, this is represented by a filled circle. The number of times that the MSE in BRR is bigger than the MSE in BSN is also shown in the plots.

Figure 4 shows scatterplots for Pearson’s correlation between observed and predicted values for individuals in the testing set obtained after fitting the BSN and BRR models for the three locations. When the correlations are higher for BSN than for BRR, this is represented by a filled circle, and by an open circle otherwise. The figure also shows the number of times Pearson’s correlation is higher for the BSN than for the BRR model. From this figure, it is clear that the BSN model predicts slightly better than the BRR model. Figure 5 shows a scatterplot for the mean squared errors in the testing set for the three locations. When the MSE in BSN is smaller than the MSE in BRR, this is represented by an open circle and by a filled circle otherwise. The number of times that the MSE in BRR is greater than the MSE in BSN is also shown in the plots. From this figure, it is clear that in general, the MSE for BRR is greater than the MSE for BSN. Table 4 shows the average Pearson’s correlation and mean squared error (MSE) between observed and predicted values in the testing set. The averages and the standard deviations are very similar for both models and the differences between the models are non-significant, but the figures suggest that the BSN model predicts slightly better than the BRR model.

**Table 4:** Average of Pearson’s correlation and mean squared error (MSE) between observed and predicted values in the testing set. The predictions were obtained after fitting the BSN and BRR models. The average is across the 100 random partitions with 80% of observations in the training set and 20% in the testing set. Standard deviations are given in parentheses

Site	Parameter		
	Model	Pearson’s correlation	MSE
Kakamega	BSN	0.2836 (0.1157)	0.7017 (0.1130)
	BRR	0.2609 (0.1163)	0.7187 (0.1212)
San Pedro Lagunillas	BSN	0.5489 (0.0895)	0.7752 (0.1031)
	BRR	0.5450 (0.0887)	0.7804 (0.1064)
Santa Catalina	BSN	0.4871 (0.1238)	0.7790 (0.1302)
	BRR	0.4804 (0.1220)	0.7685 (0.1338)

## DISCUSSION AND CONCLUSIONS

We have proposed a Bayesian regression model for skewed responses with applications when *p > >n* in the GS context, but it can also be employed in other cases and, of course, when *p < n*. In addition, to generalize linear whole genome regression models for various discrete distributions (ordinal, binomial, etc.), this study further completes the Bayesian toolbox for whole genome regression. The proposed model uses a stochastic representation of a skew normal random variable in order to facilitate the computations; it also allows using standard MCMC techniques to fit the proposed model. Results of the simulation and of applications with real data suggest that the proposed model fits the data better and also predicts slightly better than the standard Ridge Regression model. The Ridge Regression model is a particular case of our model when ρ=0. On the other hand, our results also suggest that BRR is a very robust model, although in the simulations data we already knew that it was the wrong model to fit; still, the predictive power of the model was very good. Although the conventional Bayesian whole-genome regression is robust, it does not correctly deal with skew phenotypic data, and this can decrease its genomic-enabled prediction accuracy and its goodness of fit to the data. Thus, the advantages of the proposed Bayesian whole-genome regression compensate its complexity and possible increases in computational time as compared to the conventional Bayesian ridge regression. The model proposed in this study is conceptually and operationally different, and presumably simpler than the skew-normal linear mixed model of Arellano-Valle *et al.* (2005) that uses a multivariate skew-normal distribution in order to relax normality.

Despite the fact that skewness is a major concern for breeding data analyses and may often be a result of uneven sampling of “high” and “low” performing individuals, selection, environmental effects, etc., the theoretical developments presented in this study are also applicable to many other areas of research in agronomy and in agriculture in general. For example, most crop flowering time data are indeed skewed, as well as categorical data representing different types of diseases as those presented in this research. So, skewness in phenotypical response can be the result of an artificial phenomena, the aim of this study was to propose a statistical model that will be more appropriate to deal with that problem.

Results of this study can be compared to results of two other studies, Crossa *et al.* (2011) and González-Camacho *et al.* (2012). Crossa *et al.* (2011) included one site in Mexico (San Pedro Lagunillas) that was also analyzed by transforming the original GLS ordinal scale using Box-Cox transformation; the prediction accuracy of different models (*e.g.*, Bayesian Lasso and Reproducing Kernel Hilbert Spaces) ranged from 0.416 to 0.462. Although strict comparison with the results obtained in this study is not possible because other random cross-validations were generated, the prediction accuracies of BSN (0.5489) and BRR (0.5450) models were higher than those previously reported by Crossa *et al.* (2011) for the same site.

Stochastic representation can be used to extend Reproducing Kernel Hilbert Space (de los Campos *et al.* 2010) models that in many empirical studies have led to more accurate predictions than Bayesian Ridge Regression models and Bayesian LASSO, among others (*e.g.*, Pérez-Rodríguez *et al.* 2012), so this is a topic for future research. Further studies to extend the model proposed in this study to include genotype × environment interaction should not be complicated. The proposed model can also be extended by assigning different prior distributions to the marker effects, for example, to induce variable selection. This could potentially lead to a new Bayesian alphabet with skew normal random errors.

## Supplementary Material

Supplemental material is available online at www.g3journal.org/lookup/suppl/doi:10.1534/g3.117.300406/-/DC1.

## APPENDIX A: FULL CONDITIONAL DISTRIBUTIONS

The full conditional distributions of the unknown parameters in the Bayesian Genomic Regression Model with Skew Normal Random Errors are given below. The derivation uses results from Bayesian linear models (*e.g.*, Sorensen and Gianola, 2002), as well as results from Rusell and González (2002) and Liseo and Parisi (2013).

The joint posterior distribution is as follows:

$$\begin{array}{cc} p\left(\beta_{0},\beta ,\sigma_{e}^{2},\sigma_{\beta }^{2},\rho |data\right)\propto & \left\{\overset{n}{\underset{i=1}{\prod }}\zeta \text{exp}\left\{-\frac{1}{2}\zeta^{2}{\left(y_{i}-\mu_{i}-\frac{\sigma_{e}}{S_{U}}\rho z_{i}+\frac{\sigma_{e}}{S_{U}}E_{u}\right)}^{2}-\frac{1}{2}z_{i}^{2}\right\}I_{\left(0,\infty \right)}\left(z_{i}\right)\right\}\\ & \times N\left(\beta_{0}|0,\sigma_{\beta_{0}}^{2}\right)MN_{p}\left(\beta |0,\sigma_{\beta }^{2}I\right)\chi^{-2}\left(\sigma_{e}^{2}|S_{e},df_{e}\right)\;\chi^{-2}\left(\sigma_{\beta }^{2}|S_{\beta },df_{\beta }\right)\\ & \times {\left(\frac{1-\rho }{2}\right)}^{a_{0}-1}{\left(1-\frac{1-\rho }{2}\right)}^{b_{0}-1}I_{\left(-1,1\right)}(\rho ) \end{array}$$

where $S_{U}=\sqrt{1-2/\pi \rho^{2}},\;\;E_{U}=\sqrt{2\rho /\pi },\;\;\zeta =\frac{S_{U}}{\sigma_{e}\sqrt{1-\rho^{2}}}$ and $\mu_{i}=\beta_{0}+x_{i}^{t}\beta$, **$\beta ={\left(\beta_{1},\ldots ,\beta_{p}\right)}^{t}$**.

### 1. Intercept $\left(\beta_{0}\right)$

$$\begin{array}{ccc} p\left(\beta_{0}|else\right) & \propto & \overset{n}{\underset{i=1}{\prod }}\text{exp}\left\{-\frac{1}{2}\zeta^{2}{\left(y_{i}-\beta_{0}-x_{i}^{t}\beta -\frac{\sigma_{e}}{S_{U}}\rho z_{i}+\frac{\sigma_{e}}{S_{U}}E_{U}\right)}^{2}\right\}N\left(\beta_{0}|0,\sigma_{\beta_{0}}^{2}\right)\\ & = & \overset{n}{\underset{i=1}{\prod }}N(y_{i}^{*}|\beta_{0},1/\zeta^{2})N\left(\beta_{0}|0,\sigma_{\beta_{0}}^{2}\right), \end{array}$$

where $y_{i}^{*}=y_{i}-x_{i}^{t}\beta -\frac{\sigma_{e}}{S_{U}}\rho z_{i}+\frac{\sigma_{e}}{S_{U}}E_{U}$. Note that the right-hand side of the last expression is the kernel of a normal distribution with mean $\overset{n}{\underset{i}{\sum }}y_{i}^{*}/c$ and variance $\frac{\left(1-\rho^{2}\right)\sigma_{e}^{2}}{S_{U}^{2}c}$ with $c=n+\frac{\left(1-\rho^{2}\right)\sigma_{e}^{2}}{S_{U}^{2}\sigma_{\beta_{0}}^{2}}$.

### 2. Regression coefficients $\left(\beta_{j},j=1,\ldots ,p\right)$

Here we obtain the conditional distribution of each of the elements of the vector β, *i.e.*, $p(\beta_{j}|else)$, j=1,…,p.

$$\begin{array}{ccc} p(\beta_{j}|else) & \propto & \overset{n}{\underset{i=1}{\prod }}\text{exp}\left\{-\frac{1}{2}\zeta^{2}{\left(y_{i}-\beta_{0}-x_{i}^{t}\beta -\frac{\sigma_{e}}{S_{U}}\rho z_{i}+\frac{\sigma_{e}}{S_{U}}E_{U}\right)}^{2}\right\}N\left(\beta_{j}|0,\sigma_{\beta }^{2}\right)\\ & = & \overset{n}{\underset{i=1}{\prod }}N(y_{i}^{**}|x_{ij}\beta_{j},1/\zeta^{2})N\left(\beta_{j}|0,\sigma_{\beta }^{2}\right), \end{array}$$

where $y_{i}^{**}=y_{i}-\beta_{0}-x_{i,-j}^{t}\beta_{-j}-\frac{\sigma_{e}}{S_{U}}\rho z_{i}+\frac{\sigma_{e}}{S_{U}}E_{U}$, $x_{i,-j}^{t}$ is the *i*-th row of X without the *j*-th column and $\beta_{-j}$ is the β vector without the *j*-th element. After some algebraic manipulations, it can be shown that the right-hand side of the above expression corresponds to the kernel of a normal distribution with mean $c_{2}/c_{1}$ and variance $\frac{\left(1-\rho^{2}\right)\sigma_{e}^{2}}{S_{U}^{2}c_{1}}$, with $c_{1}=\overset{n}{\underset{i=1}{\sum }}x_{ij}^{2}+\frac{\left(1-\rho^{2}\right)\sigma_{e}^{2}}{S_{U}^{2}\sigma_{\beta }^{2}}$, $c_{2}=\overset{n}{\underset{i=1}{\sum }}x_{ij}y_{i}^{**}$.

### 3. Latent variables $\left(z_{i},i=1,\ldots ,n\right)$

$$\begin{array}{ccc} p(z_{i}|else\;) & \propto & exp\left\{-\frac{1}{2(1-\rho^{2})}\left(\left(\frac{y_{i}-\mu_{i}}{\sigma_{e}}\right)S_{U}^{2}+E_{U}-\rho z_{i}\right)\right\}\text{exp}\left(-\frac{1}{2}z_{i}^{2}\right)I_{\left(0,\infty \right)}\left(z_{i}\right)\\ & = & N\left(y_{i}^{***}|\rho z_{i},1-\rho^{2}\right)\exp\left(-\frac{1}{2}z_{i}^{2}\right)I_{\left(0,\infty \right)}\left(z_{i}\right), \end{array}$$

where $y_{i}^{***}=\left(\frac{y_{i}-\mu_{i}}{\sigma }\right)S_{U}^{2}+E_{U}$. After simplifying some terms, the right-hand side of the above equation can be written as:

$$p\left(z_{i}|else\;\right)\propto exp\left\{-\frac{1}{2\left(1-\rho^{2}\right)}{\left(z_{i}-\rho y_{i}^{***}\right)}^{2}\right\}I_{\left(0,\infty \right)}\left(z_{i}\right),i=1,\ldots ,n$$.

The above expression is the kernel of a truncated normal distribution with location parameter $\rho y_{i}^{***}$ and scale parameter $1-\rho^{2}$, lower truncation bound 0 and upper truncation bound ∞. In this work, we used the R library truncnorm (Trautmann *et al.*, 2014) to sample from this distribution.

### 4. Residual variance ($\sigma_{e}^{2}$)

$$\begin{array}{ccc} p(\sigma_{e}^{2}|else) & \propto & \overset{n}{\underset{i=1}{\prod }}\frac{1}{\sigma_{e}}exp\left\{-\frac{S_{U}^{2}}{2\left(1-\rho^{2}\right)\sigma_{e}^{2}}{\left(y_{i}-\mu_{i}-\frac{\sigma_{e}}{S_{U}}\rho z_{i}+\frac{\sigma_{e}}{S_{U}}E_{U}\right)}^{2}\right\}\chi^{-2}\left(\sigma_{e}^{2}|S_{e},df_{e}\right)\\ & = & {\left(\frac{1}{\sigma_{e}}\right)}^{n}exp\left\{-\frac{S_{U}^{2}}{2\left(1-\rho^{2}\right)\sigma_{e}^{2}}\overset{n}{\underset{i=1}{\sum }}{\left(y_{i}-\mu_{i}-\frac{\sigma_{e}}{S_{U}}\rho z_{i}+\frac{\sigma_{e}}{S_{U}}E_{U}\right)}^{2}\right\}\\ & \times & {\left(\sigma_{e}^{2}\right)}^{-\left(\frac{df_{e}}{2}+1\right)}exp\left\{-\frac{S_{e}}{2\sigma_{e}^{2}}\right\}\\ & = & {\left(\sigma_{e}^{2}\right)}^{-\left(\frac{n+df_{e}}{2}+1\right)}exp\left\{-\frac{1}{2\sigma_{e}^{2}}\left[S_{e}+\frac{S_{U}^{2}}{\left(1-\rho^{2}\right)}\overset{n}{\underset{i=1}{\sum }}{\left(y_{i}-\mu_{i}-\frac{\sigma_{e}}{S_{U}}\rho z_{i}+\frac{\sigma_{e}}{S_{U}}E_{U}\right)}^{2}\right]\right\}, \end{array}$$

Note that $p(\sigma_{e}^{2}|else)$ is a very complex function of $\sigma_{e}^{2}$ and its kernel does not correspond to any known univariate density function, so we have to sample this parameter by Metropolis algorithm or another MCMC technique. Following Hea-Jung (2005), we considered the Random Walk Metropolis Algorithm with a de-constraint transformation to sample $\sigma_{e}^{2}$. Given the fact that $\sigma_{e}^{2}>0,$ let $\xi =\text{log}(\sigma_{e}^{2})$ so that ξ has support in ℝ. Note that the density function of ξ can be obtained by using the transformation method (Casella and Berger, 2002, Chapter 2), which is given by:

$$p\left(\;\xi |else\right)\propto p\left(\sigma_{e}^{2}|else\right)\times \text{exp}(\xi )$$.

In the Random Walk Metropolis Algorithm, we generated ξ by choosing a proposal transition kernel to add noise to the current state. Assuming that the actual value of ξ is $\xi_{k}$, and that we wanted to update its value so that in the next iteration we had $\xi_{k+1}$, we followed steps a-c below.

a. Sample ξ, $\xi =\xi_{k}+Z$, where $Z∼N\left(0,\eta^{2}\right)$.

b. Sample u, U∼Uniform(0,1).

c. If $u<p\left(\;\xi |else\right)/p\left(\;\xi_{k}|else\right),$ then $\xi_{k+1}=\;\xi$; otherwise $\xi_{k+1}=\xi_{k}$.

Once $\xi_{k+1}$ has been obtained, compute $\sigma_{e,k+1}^{2}=\text{exp}(\xi_{k+1})$. The parameter $\eta^{2}$ can be modified so that we have an optimal acceptation rate (Roberts *et al.*, 1997).

### 5. Variance of markers ($\sigma_{\beta }^{2}$)

$$\begin{array}{ccc} p\left(\sigma_{\beta }^{2}|else\right) & \propto & NM_{p}\left(\beta |0,\sigma_{\beta }^{2}I\right)\times \;\chi^{-2}\left(\sigma_{\beta }^{2}|S_{\beta },df_{\beta }\right)\\ & = & {\left(\sigma_{\beta }^{2}\right)}^{-p}exp\left\{-\frac{1}{2\sigma_{\beta }^{2}}\beta^{t}\beta \right\}{\left(\sigma_{\beta }^{2}\right)}^{-\left(\frac{df_{\beta }}{2}+1\right)}exp\left\{-\frac{S_{\beta }}{2\sigma_{\beta }^{2}}\right\}\\ & = & {\left(\sigma_{\beta }^{2}\right)}^{-\left(\frac{p+df_{\beta }}{2}+1\right)}exp\left\{-\frac{1}{2\sigma_{\beta }^{2}}\left[\beta^{t}\beta +S_{\beta }\right]\right\}\\ & = & \;\chi^{-2}\left(\sigma_{\beta }^{2}|\beta^{t}\beta +S_{\beta },df_{\beta }+p\right) \end{array}$$

### 6. Correlation coefficient (ρ)

$$\begin{array}{ccc} p(\rho |else) & \propto & \overset{n}{\underset{i=1}{\prod }}\frac{S_{U}}{\sqrt{1-\rho^{2}}}exp\left\{-\frac{S_{U}^{2}}{2\left(1-\rho^{2}\right)\sigma_{e}^{2}}{\left(y_{i}-\mu_{i}-\frac{\sigma_{e}}{S_{U}}\rho z_{i}+\frac{\sigma_{e}}{S_{U}}E_{U}\right)}^{2}\right\}\\ & \times & {\left(\frac{1-\rho }{2}\right)}^{a_{0}-1}{\left(1-\frac{1-\rho }{2}\right)}^{b_{0}-1}I_{\left(-1,1\right)}(\rho )\\ & = & {\left(S_{U}\right)}^{n}{\left(1-\rho^{2}\right)}^{-n/2}exp\left\{-\frac{S_{U}^{2}}{2\left(1-\rho^{2}\right)\sigma_{e}^{2}}\overset{n}{\underset{i=1}{\sum }}{\left(y_{i}-\mu_{i}-\frac{\sigma_{e}}{S_{U}}\rho z_{i}+\frac{\sigma_{e}}{S_{U}}E_{U}\right)}^{2}\right\}\\ & \times & {\left(\frac{1-\rho }{2}\right)}^{a_{0}-1}{\left(1-\frac{1-\rho }{2}\right)}^{b_{0}-1}I_{\left(-1,1\right)}(\rho )\; \end{array}$$

Note that p(ρ|else) is a very complex function of ρ and its kernel does not correspond to any known univariate density function, so we must obtain samples using the Metropolis algorithm or another MCMC technique. Here we propose using Fisher’s (1915) transformation of ρ defined as $\vartheta =\frac{1}{2}\log\left(\frac{1+\rho }{1-\rho }\right)=\tanh^{-1}(\rho )$, so that ϑ has support in ℝ. Note that the density function of ϑ can be obtained using the transformation method (Casella and Berger, 2002, Chapter 2), which is given by

$$p\left(\;\vartheta |else\right)\propto p\left(\rho |else\right)\times {\text{sech}}^{2}(\vartheta )$$.

In the Random Walk Metropolis Algorithm, we generated ϑ by choosing a proposal transition kernel to add noise to the current state. Assuming that the actual value of ϑ is $\vartheta_{k}$ and that we wanted to update its value so that in the next iteration we had $\vartheta_{k+1}$, we followed steps d-f below.

d. Sample ϑ, $\vartheta =\vartheta_{k}+Z$, where $Z∼N\left(0,\nu^{2}\right)$.

e. Sample u, U∼Uniform(0,1).

f. If $u<p\left(\;\vartheta |else\right)/p\left(\;\vartheta_{k}|else\right)$, then $\vartheta_{k+1}=\;\vartheta$; otherwise $\vartheta_{k+1}=\vartheta_{k}$.

Once $\vartheta_{k+1}$ has been obtained, compute $\rho =\tanh(\vartheta_{k+1})$. The parameter $\nu^{2}$ can be modified so that we have an optimal acceptation rate (Roberts *et al.*, 1997).

The samples from the posterior distribution can be obtained using the Gibbs Sampler (Geman and Geman, 1984) and the Random Walk Metropolis algorithm. In the algorithm, we sampled each of the fully conditional distributions until we obtained a sample of the desired size. We implemented the algorithm in the R Statistical package (R Core Team, 2016). In order to speed up computations, the routines that sample from $p(\beta_{j}|else)$, j=1,…,p were implemented in C programming language (Kernighan and Ritchie, 1988), a shared library was generated and then the compiled routines were used in R. The R script and the C source code are available upon request from the first author.

## APPENDIX B: SETTING THE HYPER-PARAMETERS FOR THE PRIOR DISTRIBUTIONS

The hyper-parameters can be set using a set of default rules similar to those used in the BGLR software (de los Campos *et al.*, 2013; Pérez and de los Campos 2014). With these rules we assigned proper but weakly informative prior distributions so that we partitioned the total variance of the phenotypes into two components: (1) the error and (2) the linear predictor, that is:

$$\begin{array}{ccc} Var(y_{i}) & = & Var\left(\overset{p}{\underset{j=1}{\sum }}x_{ij}\beta_{j}+e_{i}\right)\\ & = & Var\left(\overset{p}{\underset{j=1}{\sum }}x_{ij}\beta_{j}\right)+Var(e_{i})\\ & = & \overset{p}{\underset{j=1}{\sum }}x_{ij}^{2}Var(\beta_{j})+Var(e_{i})\\ & = & \sigma_{\beta }^{2}\overset{p}{\underset{j=1}{\sum }}x_{ij}^{2}+\sigma_{e}^{2}=Var\left(g_{i}\right)+Var(e_{i}) \end{array}$$ (B.1)

where $g_{i}=\overset{p}{\underset{j=1}{\sum }}x_{ij}\beta_{j}$. A priori, the total genetic variance is $\overset{n}{\underset{i=1}{\sum }}Var\left(g_{i}\right)=\sigma_{\beta }^{2}\overset{n}{\underset{i=1}{\sum }}\overset{p}{\underset{j=1}{\sum }}x_{ij}^{2}$ and the a priori average genetic variance is:

$$V_{g}=\frac{\sigma_{\beta }^{2}}{n}\overset{n}{\underset{i=1}{\sum }}\overset{p}{\underset{j=1}{\sum }}x_{ij}^{2}=\sigma_{\beta }^{2}MSx,$$ (B.2)

where $MSx=\frac{1}{n}\overset{n}{\underset{i=1}{\sum }}\overset{p}{\underset{j=1}{\sum }}x_{ij}^{2}$. So from (B.1) and (B.2), the partition of the phenotypic variance is given by:

$$V_{y}=V_{g}+V_{e}=\sigma_{\beta }^{2}MSx+\sigma_{e}^{2},$$ (B.3)

where $V_{e}=Var\left(e_{i}\right)=\sigma_{e}^{2}$.

### Setting the hyper-parameters for $\;\chi^{-2}\left(\sigma_{\beta }^{2}|S_{\beta },df_{\beta }\right)$

In the parametrization used in this work, $E\left(\sigma_{\beta }^{2}|S_{\beta },df_{\beta }\right)=\frac{S_{\beta }}{df_{\beta }-2}$ and $Mode\left(\sigma_{\beta }^{2}|S_{\beta },df_{\beta }\right)=\frac{S_{\beta }}{df_{\beta }+2}$. We set $df_{\beta }$=5 so that the prior distribution has a finite mean. From equation (B.2),

$$\sigma_{\beta }^{2}=\frac{V_{g}}{MSx}$$, (B.4)

thus, if we replace the left-hand side of (B.4) with the mode of $\sigma_{\beta }^{2}|S_{\beta },df_{\beta }$ then:

$$\frac{S_{\beta }}{df_{\beta }+2}=\frac{V_{g}}{MSx\;}$$. (B.5)

From (B.5) and solving for $S_{\beta }$, we obtain $S_{\beta }=\frac{V_{g}\times \left(df_{\beta }+2\right)}{MSx}$. From the definition of heritability, $h^{2}=V_{g}/V_{y}$, so that $V_{g}=h^{2}V_{y}$; then:

$$S_{\beta }=\frac{h^{2}V_{y}\times \left(df_{\beta }+2\right)}{MSx}=\frac{R^{2}V_{y}\times \left(df_{\beta }+2\right)}{MSx}$$ (B.6)

Once we set $df_{\beta },$ we can set $S_{\beta }$, and we only need to compute the phenotypic variance $\left(V_{y}\right)$, MSx and set $R^{2}$ as the proportion of the variance that is explained a priori by the markers. By default we set $R^{2}=0.5$.

### Setting the hyper-parameters for $\;\chi^{-2}\left(\sigma_{e}^{2}|S_{e},df_{e}\right)$

In the parametrization used in this work, $E\left(\sigma_{e}^{2}|S_{e},df_{e}\right)=\frac{S_{e}}{df_{e}-2}$ and $Mode\left(\sigma_{e}^{2}|S_{e},df_{e}\right)=\frac{S_{e}}{df_{\beta }+2}$,

we set $df_{e}=5$ and $S_{e}=(1-R^{2})V_{y}\times \left(df_{e}+2\right)$.

### Setting the hyper-parameters for $N\left(\beta_{0}|0,\sigma_{\beta_{0}}^{2}\right)$

We set $\sigma_{\beta_{0}}^{2}=1\times 10^{6}$ so that the prior assigned to the intercept is effectively a flat one.

### Setting the hyper-parameters for $p(\rho |a_{0},b_{0})$

The density function of the prior assigned to ρ is:

$$p\left(\rho |a_{0},b_{0}\right)=\frac{1}{2Beta\left(a_{0},b_{0}\right)}{\left(\frac{1-\rho }{2}\right)}^{a_{0}-1}{\left(1-\frac{1-\rho }{2}\right)}^{b_{0}-1}I_{\left(-1,1\right)}(\rho ),a_{0}>0,\;b_{0}>0$$

Figure B.1 shows the density function of ρ for different values of the parameters $a_{0},b_{0}$. Setting different values for those hyper-parameters could lead to a rich variety of shapes, just as in the Beta distribution. Note that if we set $a_{0}=b_{0}=1$, then we obtain a Uniform(−1,1) prior that corresponds to the one used in this work.
